# Bromelain inhibits SARS‐CoV‐2 infection via targeting ACE‐2, TMPRSS2, and spike protein

**DOI:** 10.1002/ctm2.281

**Published:** 2021-01-17

**Authors:** Satish Sagar, Ashok Kumar Rathinavel, William E. Lutz, Lucas R. Struble, Surender Khurana, Andy T. Schnaubelt, Nitish Kumar Mishra, Chittibabu Guda, Nicholas Y. Palermo, Mara J. Broadhurst, Tobias Hoffmann, Kenneth W. Bayles, St. Patrick M. Reid, Gloria E. O. Borgstahl, Prakash Radhakrishnan

**Affiliations:** ^1^ Eppley Institute for Research in Cancer and Allied Diseases University of Nebraska Medical Center Omaha Nebraska USA; ^2^ Division of Viral Products Center for Biologics Evaluation and Research (CBER) FDA Silver Spring Maryland USA; ^3^ Department of Pathology and Microbiology University of Nebraska Medical Center Omaha Nebraska USA; ^4^ Department of Genetics Cell Biology and Anatomy University of Nebraska Medical Center Omaha Nebraska USA; ^5^ Fred & Pamela Buffett Cancer Center University of Nebraska Medical Center Omaha Nebraska USA; ^6^ Computational Chemistry Core University of Nebraska Medical Center Omaha Nebraska USA; ^7^ Advanced Light and Electron Microscopy Centre for Biological Threats and Special Pathogens 4 (ZBS 4) Robert Koch Institute Berlin Germany

Dear Editor,

The new coronavirus, SARS‐CoV‐2, transmits rapidly from human‐to‐human resulting in the ongoing pandemic. SARS‐CoV‐2 infects angiotensin‐converting enzyme 2 (ACE‐2) expressing lung, heart, kidney, intestine, gall bladder, and testicular tissues of patients, leading to organ failure and sometimes death.^1^, ^2^ Currently, COVID‐19 patients are treated with different agents, including favilavir, remdesivir, chloroquine, hydroxychloroquine, lopinavir, darunavir, and tocilizumab.^3^, ^4^ However, the safety and efficacy of those drugs against COVID‐19 still need further confirmation by randomized clinical trials. Hence, there is an emergent need to repurpose the existing drugs or develop new virus‐based and host‐based antivirals against SARS‐CoV‐2. Bromelain is a cysteine protease isolated from pineapple stem and is used as a dietary supplement for treating patients with pain, inflammation,^5^ thrombosis,^6^ and cancer.^7^

Recently, studies have shown that SARS‐CoV‐2 homotrimeric viral spike protein (S1) binds to the Transmembrane Serine Protease 2 (TMPRSS2) primed host cell's receptor ACE‐2 for initial entry, followed by S2‐mediated membrane fusion.^8^ Of several normal and cancerous cells tested, VeroE6 and Calu‐3 cells showed ACE‐2 protein expression (Fig. 1A), as well as a basal level of TMPRSS2 protein (Fig. 1B). Since ACE‐2^9^ and TMPRSS2 (UniProtKB‐O15393) contains cysteine residues with disulfide bonds to stabilize the protein structure, we investigated the effect of bromelain on ACE‐2 and TMPRSS2 expression. Bromelain‐induced a dose‐ and time‐dependent reduction of ACE‐2 and TMPRSS2 expression in VeroE6 cells (Fig. 1C and D) but do not alter ACE‐2 expression in Calu‐3 cells (Fig. 1E). However, bromelain reduces the expression of TMPRSS2 in Calu‐3 (Fig. 1E) and ACE‐2 negative normal bronchial epithelial (BEAS‐2B) and lung adenocarcinoma (A549) cells (Fig. 1F and G). Cysteine protease inhibitor (E‐64) treatment further confirmed that bromelain's cysteine protease activity could cleave/reduce the expression of ACE‐2 and TMPRSS2 (Fig. 1H). Surface plasmon resonance (SPR) analysis revealed that purified SARS‐CoV‐2 S‐ectodomain binds with ACE‐2 in a concentration‐dependent manner and has a comparable binding affinity as control RBD (Fig. 1I). The calculated molecular weight of the purified S‐ectodomain‐GFP protein is ∼165 kDa; however, we observed a higher molecular weight of S‐ectodomain (∼215 kDa), which may be due to heavy N‐ and O‐linked glycosylation (Fig. 1I intent). A serological assay showed a significantly increased median fluorescent intensity (MFI) of purified S‐ectodomain with COVID‐19 positive patients’ samples (Fig. 1J). These two results indicated that purified S‐ectodomain is a properly folded and functionally active protein.

**FIGURE 1:**
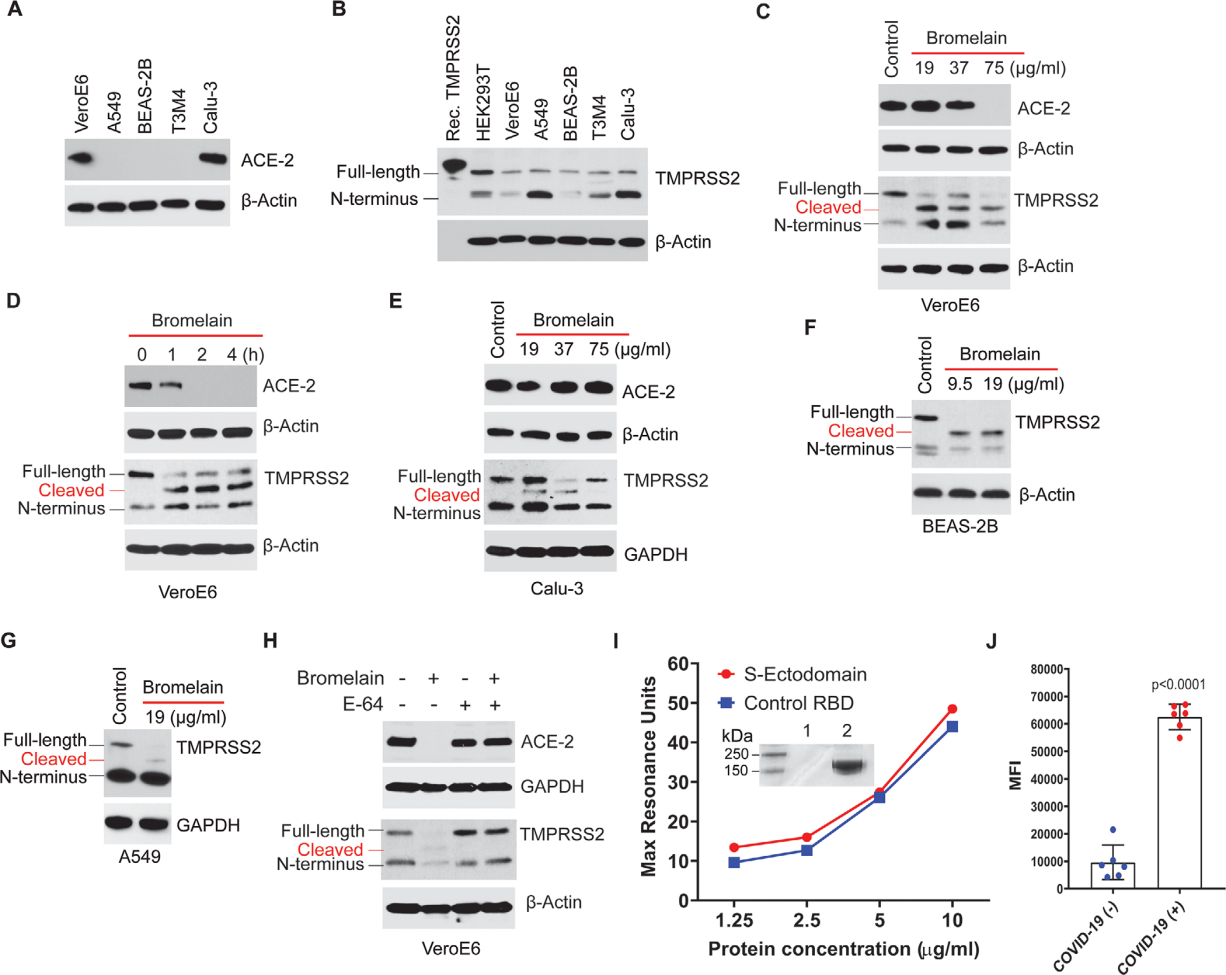
Bromelain inhibits ACE‐2 and TMPRSS2 expression. (A and B) Immunoprobing of ACE‐2 and TMPRSS2 expression in various normal and cancerous cells. (C and D) Immunoblotting of ACE‐2 and TMPRSS2 in VeroE6 cells treated with varying dose (19, 37, and 75 µg/ml for 48 h) and time (75 µg/ml for 1–4 h), respectively. (E) Immunoblotting of ACE‐2 and TMPRSS2 in Calu‐3 cells treated with varying dose (19, 37, and 75 µg/ml for 48 h). (F and G) Immunoblotting of TMPRSS2 in BEAS‐2B and A549 cells treated with varying dose (9.5 and 19 µg/ml for 48 h). (H) ACE‐2 and TMPRSS2 expression in bromelain (75 µg/ml) plus E‐64 (4 µM) treated VeroE6 cells. β‐Actin and GAPDH served as a loading control. (I) SPR Max Resonance Units (RSU) at equilibrium as a function of the concentration of control RBD and S‐ectodomain using immobilized ACE‐2. S‐ectodomain‐eGFP purified from Tni insect cells and showed ∼215 kDa molecular weight (intent; 1, mock control; 2, S‐ectodomain‐eGFP). (J) Luminex assay median fluorescent intensity (MFI) of S‐ectodomain with COVID‐19 positive (*n* = 6) and negative (*n* = 6) patients’ samples.

The S‐ectodomain has 30 cysteine amino acids with 15 stabilizing disulfide bonds (UniProtKB: P0DTC2) (Fig. 2A). The RBD domain alone has nine cysteine residues, eight of which form four disulfide linkages. Bromelain‐induced a dose‐ and time‐dependent cleavage of S‐ectodomain in Tni insect cell supernatant (Fig. 2B and C) and purified S‐ectodomain (Fig. 2D). Heat inactivation and cysteine proteinase inhibitor (E‐64) treatment inhibited bromelain mediated digestion of S‐ectodomain (Fig. 2E and F). Further, SARS‐CoV‐2 with bromelain treatment showed the loss of Spike protein on the viral surface (Fig. 2G). Our docking studies between homotrimeric (RBD containing chain A) S protein and stem bromelain revealed that bromelain cleaves the S‐protein equally likely at the 131–166 (50.4%) and 617–649 (49.6%) disulfide bonds (Fig. 2H). Though the catalytic site is not directly engaged, protein–protein docking places the enzyme in close proximity to these bonds. These results demonstrate that bromelain's cysteine protease activity is responsible for the cleavage of host cells’ ACE‐2 and SARS‐CoV‐2 S‐protein.

**FIGURE 2:**
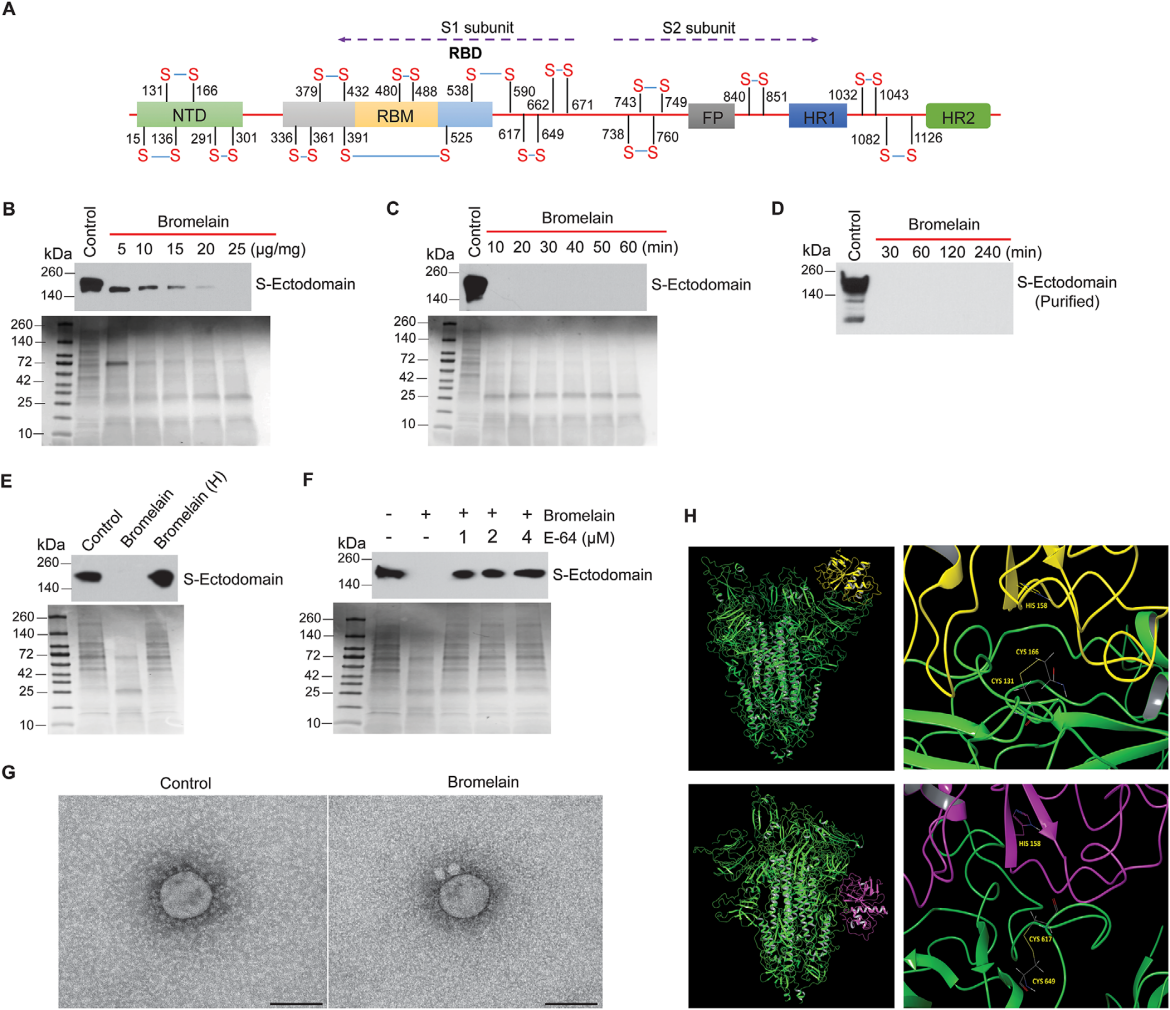
Bromelain cleaves SARS‐CoV‐2 S‐ectodomain. (A) Schematic representation of predicted cysteine amino acid position and disulfide bridges in SARS‐CoV‐2 S‐ectodomain. (B and C) Immunoblotting of S‐ectodomain in bromelain‐treated Tni insect cell supernatant varying dose (5, 10, 15, 20, and 25 µg/mg of total protein) and time (25 µg/mg of total protein for 10–60 min), respectively. (D) Immunoprobing of bromelain‐treated purified S‐ectodomain (1:10 ratio) with 30, 60, 120, and 240 min. (E and F) Immunoprobing of S‐ectodomain in heat‐inactivated bromelain (80°C/8 min) and bromelain (25 µg/mg of total protein) plus E‐64 (1, 2, and 4 µM) treated Tni supernatant, respectively. SimplyBlue‐stained gel images of bromelain‐treated Tni supernatant served as a loading control. (G) Negative‐staining transmission‐EM of bromelain‐treated (250 µg /ml, at 37°C for 1.5 h) SARS‐CoV‐2. Viral particles treated with vehicle control (left) and bromelain (right). Scale bar = 100 nm. Experiments were performed thrice, and a representative image is presented. (H) Representative conformation of bromelain (yellow and magenta) docked with the S protein (green) near the 131–166 (upper‐left; closer view upper right) and 617–649 (lower‐left; closer view lower right) disulfide bridge

Since bromelain digested ACE‐2 and S‐ectodomain, we investigated the effect of bromelain on the interactions of S‐ectodomain and SARS‐CoV‐2 with VeroE6 cells. Bromelain significantly reduced the binding of S‐protein to VeroE6 cells (Fig. 3A and B) and was further confirmed by cysteine protease inhibitor (E‐64) treatment (Fig. 3C). Interestingly, bromelain pre‐treatment significantly decreased SARS‐CoV‐2 viral binding in VeroE6 cells (*P* = .0021) (Fig. 3D). Most importantly, VeroE6 cells or SARS‐CoV‐2 or both with bromelain reduces the viral infection (Fig. 3E and F). Additionally, we found significantly reduced SARS‐CoV‐2 viral RNA copies in bromelain‐treated VeroE6 (*P* = .0010) and Calu‐3 (*P* = .0099) cells (Fig. 3G and H, respectively). Collectively, these results suggest that bromelain could inhibit SARS‐CoV‐2 binding and infection in VeroE6 and Calu‐3 cells. Studies have demonstrated that SARS‐CoV‐2 S‐protein has high homology among other coronaviruses (76% identity with SARS‐CoV) with conserved cysteine amino acids (UniProtKB: P59594). This indicates that bromelain may be used as a broad antiviral agent against SARS‐CoV‐2 and other related family members.

**FIGURE 3:**
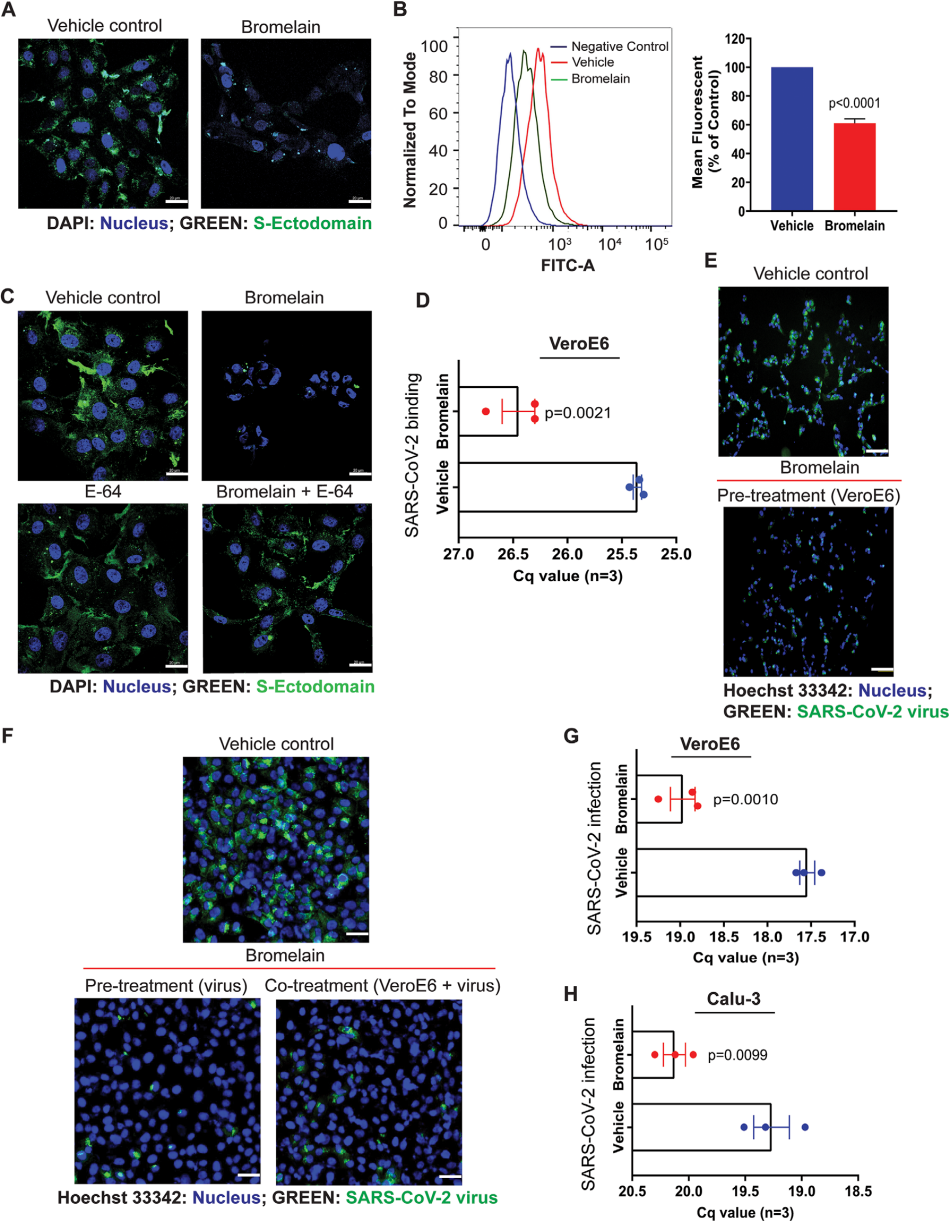
Bromelain inhibits SARS‐CoV‐2 binding and infection. (A and B) Immunofluorescence and flow cytometry analysis of S‐ectodomain in vehicle‐ (PBS) and bromelain (75 µg/ml/2 h)‐treated VeroE6 cells (*n* = 3), respectively. The mean fluorescent intensity was measured by using FlowJo software. Experiments were performed twice, and one set of representative data is presented. Mean ± SD. (C) Immunofluorescence of S‐ectodomain in‐vehicle, bromelain (75 µg/ml) with and without E‐64 (4 µM)‐treated VeroE6 cells (*n* = 3) for 2 h. Nuclei were stained with DAPI. Scale bar = 20 µm. (D) qRT‐PCR analysis of SARS‐CoV‐2 RNA in vehicle and bromelain pre‐treated VeroE6 cells. The Cq values were graphically represented. Mean ± SD (*n* = 3). Immunofluorescence analysis of SARS‐CoV‐2 in bromelain pre‐treated VeroE6 cells (75 µg/ml/2 h) (*n* = 6) (E), bromelain pre‐treated SARS‐CoV‐2 (75 µg/ml/1 h) and bromelain co‐treated SARS‐CoV‐2 plus VeroE6 cells (75 µg/ml/1 h) (*n* = 4) (F). Scale bar = 50 µm. Nuclei were stained with Hoechst 33342. (G and H) qRT‐PCR analysis of SARS‐CoV‐2 RNA infected bromelain‐treated VeroE6 and Calu‐3 cell culture supernatants, respectively (*n* = 3). The Cq values were graphically represented. Mean ± SD (*n* = 3). *P* < .05 was considered statistically significant with an unpaired Student's *t*‐test

In conclusion, the currently used drugs against SARS‐CoV‐2 have potential side effects. Vaccine trials have started against COVID‐19, but the host immune response against SARS‐CoV‐2 is not fully understood. It differs between individuals, and also re‐infection of individuals with SARS‐CoV‐2. For the first time, our results demonstrate that bromelain can inhibit SARS‐CoV‐2 infection via targeting ACE‐2, TMPRSS2, and SARS‐CoV‐2 S‐protein. Also, thrombosis development is a significant risk factor of multiorgan failure and death in COVID‐19 patients.^10^ Since bromelain inhibits SARS‐CoV‐2 infection, and its profound fibrinolytic activity ^6^ suggests that bromelain or bromelain‐rich pineapple could be used as an antiviral against SARS‐CoV‐2 and future outbreaks of other coronaviruses.

## CONFLICT OF INTEREST

Satish Sagar and Prakash Radhakrishnan have ownership interest (including patents) in a pending patent.

## ETHICS APPROVAL AND CONSENT TO PARTICIPATE

The institutional review board (IRB) of the University of Nebraska Medical Center approved clinical samples used in this study.

## AUTHOR CONTRIBUTIONS

Prakash Radhakrishnan conceived the idea; Surender Khurana, St. Patrick M. Reid, Gloria E. O. Borgstahl, and Prakash Radhakrishnan designed research; Satish Sagar, Ashok Kumar Rathinavel, William E. Lutz, Lucas R. Struble, St. Patrick M. Reid, Tobias Hoffmann, and Mara J. Broadhurst performed research; Nitish Kumar Mishra, Chittibabu Guda, Nicholas Y. Palermo, and Kenneth W. Bayles contributed new reagents/analytic tools; Satish Sagar, Ashok Kumar Rathinavel, Andy T. Schnaubelt, Gloria E. O. Borgstahl, St. Patrick M. Reid, and Prakash Radhakrishnan analyzed data; Gloria E. O. Borgstahl, and Prakash Radhakrishnan wrote the paper.

## DATA AVAILABILITY STATEMENT

Materials are available upon a reasonable request from the corresponding author.

## Supporting information

Detailed materials and methods and key resources are included in the supplementary information.
