# Supplementary material for: Bromelain inhibits SARS‐CoV‐2 infection via targeting ACE‐2, TMPRSS2, and spike protein
Source: Clin Transl Med. 2021 Jan 17;11(2):e281. doi: 10.1002/ctm2.281 (PMC7811777; doi:10.1002/ctm2.281)
Supplement: Supplementary file 1 — Detailed materials and methods and key resources are included in the supplementary information. [file CTM2-11-e281-s001.docx]

**Bromelain inhibits SARS-CoV-2 infection via targeting ACE-2, TMPRSS2 and spike protein**

Satish Sagar^1^, Ashok Kumar Rathinavel^1^, William E. Lutz^1^, Lucas R. Struble^1^, Surender Khurana^2^, Andy T Schnaubelt^3^, Nitish Kumar Mishra^4^, Chittibabu Guda^4,5^, Nicholas, Y. Palermo^6^, Mara J. Broadhurst^3^, Tobias Hoffmann^7^, Kenneth W. Bayles^3^, St. Patrick M. Reid^3^, Gloria E. O. Borgstahl^1, 5^ and Prakash Radhakrishnan^1, 3, 4, 5^ *

**Materials and Methods**

**Cells and cell culture conditions**

The African green monkey kidney epithelial cells (VeroE6, ATCC, VA, USA), human lung adenocarcinoma cells (A549, a kind gift from Maher Abdulla, Department of Pathology and Microbiology, UNMC and Calu-3, a kind gift from John Dickinson, Internal Medicine Division of Pulmonary, Critical Care and Sleep, UNMC), human normal bronchial epithelial cells (BEAS-2B, a kind gift from Todd Wyatt, Internal Medicine Division of Pulmonary, Critical Care and Sleep, UNMC), human Pancreatic cancer cells (T3M4, a kind gift from Michael A Hollingsworth, Eppley Institute for Research in Cancer, UNMC), and human embryonic kidney epithelial cells (HEK293T, ATCC, VA, USA) were used for this study. VeroE6, A549, T3M4, and HEK293T were grown in DMEM (Gibco, MA, USA) with 10% Fetal bovine serum (FBS, Corning, MA, USA) and 1X penicillin and streptomycin (Corning, MA, USA). Calu-3 cells were grown in EMEM (ATCC, VA, USA) with 10% FBS and 1X penicillin and streptomycin. BEAS-2B cells were grown in DMEM with 5% FBS and 1X penicillin and streptomycin. All the cells were maintained at 37°C with 5% CO2 in a humidified chamber.

**Virus and culture conditions**

SARS-CoV-2 strain (BEI_USA-WA1/2020) was obtained from the BEI and propagated in VeroE6 cells. All the experiments and the viral titer (by plaque assay) were performed in the BSL3 laboratory at the University of Nebraska Medical Center, Omaha, USA.

**Recombinant S-Ectodomain-GFP baculovirus preparation**

The full-length genome sequences of 45 SARS-CoV-2 isolates were analyzed. Bat Coronavirus genome annotations were used to map the spike protein on the Wuhan CoVs. Multiple sequence alignment for 45 spike proteins was carried out, and the full-length consensus sequence was derived using the Gene Calc algorithm. The membrane-spanning region was identified using DNAstar Protean software. The cDNA for SARS-CoV-2 Spike protein ectodomain residues 1 to 1220 (S-Ectodomain-GFP) was chemically synthesized with optimal insect cell codons (Genscript USA Inc, NJ, USA) and cloned into pVL1393 (Expression Systems, CA, USA) with an insect cell secretion signal. This construct includes complete S1 and S2 regions and ends just before the transmembrane region. The furin cleavage site is mutated from RRAR to GSAS *(1)*. Tobacco Etch Virus (TEV) protease cleavage site was added to the C-terminus, followed by a flexible linker, eGFP, and 12X histidine tag. A recombinant baculovirus (P0) was produced in SF9 cells (Expression Systems, CA, USA) as described in the protocol of the BestBac^tm^ 2.0 Δ v-cath/ChiA Baculovirus Co-transfection Kit (Expression Systems, CA, USA). P1 baculovirus was generated with suspended Sf9 cells (2 x10^6^ cells/ml) with a multiplicity of infection (MOI: number of virus particles/number of cells) of 0.1. Viral titer was measured using a flow cytometric assay for gp64 expression (Expression Systems, CA, USA).

**Recombinant S-Ectodomain-GFP protein expression and purification**

Tni cells (2x10^6^ cells/ml) suspended in 1L of ESF-AF media at were infected with the P1 virus at an MOI of 5. Cells were harvested 72 hours post-infection by centrifugation (500xg, 15 minutes). One cOmplete^TM^ ULTRA protease inhibitor tablet (Roche, MA, USA) per liter was added before centrifugation. The supernatant was concentrated by tangential flow filtration (TFF) using a Pellicon® 2 mini-cassette system (Millipore, MO, USA) with a 30 kDa pore size. The concentrated supernatant was adjusted to 40 mM TRIS-HCl and 1 M sodium chloride. Then the pH was adjusted to 8.0 with sodium hydroxide and was then centrifuged (14,500xg, 30 minutes) and sterile filtered using a Thermo Scientific^TM^ Nalgene^TM^ Rapid-Flow^TM^ 90 mM Filter Unit-1000 ml. For affinity purification, 10 ml of Ni Sepharose excel resin (Cytiva life sciences, USA) was added for every 1 L of culture (pre-TFF volume) and incubated overnight, gently stirring at 4°C. Stirring was stopped to allow the resin to settle for 1 hour, and then the supernatant was decanted, the remaining resin and media were mixed well and poured into a gravity flow column (Bio-rad, Hercules, CA, USA). The column was treated with 3 column volumes (CVs) of wash buffer (40 mM TRIS-HCl pH 8 with 1M sodium chloride), 3 CV of wash buffer with 20 mM imidazole added, 3 CV of wash buffer with 40 mM imidazole, and finally 3 CV of wash buffer with 60 mM imidazole before protein elution with 3 CV of wash buffer with 500 mM imidazole. S-Ectodomain-GFP protein was concentrated with an Amicon® Ultra 15 Centrifugal Filter (100 kDa MWCO). It was injected onto a HiLoad^TM^ 16/600 Superdex^TM^ 200 prep grade FPLC size-exclusion column using 1x PBS (Fisher scientific, MA, USA) as a running buffer. The peaks were collected with 1 mL fractions. Pure fractions were identified by SDS-PAGE (MW ~250 kDa), pooled, and concentrated to 0.5 mg/ml.

**Surface Plasmon Resonance (SPR) based hACE2 binding assay**

The recombinant hACE2-AviTag protein (Acro Biosystems, DE, USA) was captured on a sensor chip in the test flow channels. Samples of 300 μl of freshly prepared serial dilutions of the purified recombinant protein were injected at a flow rate of 50 μl/min (contact duration 180 seconds) for the association. Responses from the protein surface were corrected for the response from a mock surface and for responses from a buffer-only injection. Total hACE2 binding and data analysis were calculated with Bio-Rad ProteOn Manager software (version 3.1).

**Luminex serological assay**

This assay was performed using the purified SARS-CoV-2 S-Ectodomain (5 μg) coupled to the surface of group A, region 43, MagPlex® Microspheres (Luminex Corp, IL, USA). Microsphere coupling was performed using the Luminex xMAP Antibody Coupling Kit (Luminex Corp, IL, USA) according to the manufacturer’s instructions. The protein-coupled microspheres were re-suspended in PBS-TBN buffer (1X PBS containing 0.1% Tween 20, 0.5% BSA and 0.1% sodium azide) at a final stock concentration of 2X10^6^ microspheres per ml, with 2.5X10^3^ microspheres used per reaction. The serum samples were obtained from de-identified COVID-19 patients (n=6) and healthy donors (n=6) at UNMC. All human samples were collected under an Institutional Review Board approved protocol (IRB PROTOCOL# 146-20-FB) by UNMC. Fifty microliters of each serum sample (diluted 1:25 with 1X PBS-TBN buffer) was mixed with 50 μl of the S-Ectodomain coupled microspheres in a 96 well-plate (Greiner Bio-One). The assay plate was incubated for 30 minutes at 37°C with shaking at 700 RPM and then washed 5 times with 1X PBS-TBN buffer. Then, the plate was incubated with biotin conjugated goat anti-human IgG (Abcam, MA, USA), labeled with streptavidin R-phycoerythrin reporter (Luminex xTAG® SA-PE G75) for 1 h at 25°C with shaking at 700RPM. Then, the plate was washed 5 times with 1X PBS-TBN buffer. Finally, the plate was re-suspended in 100 μl of 1X PBS-TBN buffer and incubated for 10 minutes at 25°C with shaking at 700 RPM. The microplate was assayed on a Luminex MAGPIX^TM^ System, and results were reported as median fluorescent intensity (MFI).

**Bromelain treatment**

VeroE6 cells were grown in 10% FBS containing media and treated with bromelain (Sigma-Aldrich, MA, USA) at different concentrations (9.5, 19, 37, and 75 μg/ml) for 48 h. For the time-dependent study, the aforementioned cells were treated with bromelain (75 μg/ml) in a serum-free condition for 0-4 h. After the end of the treatment, the cells were washed and lysed with RIPA lysis buffer (ThermoFisher, MA, USA) containing protease inhibitors (Roche, MA, USA). SARS-CoV-2 S-Ectodomain expressing Tni cell media were treated with different concentrations of bromelain (5, 10, 15, 20, and 25 μg) for 1 mg of total protein at 37^°^C for 1h. For the time-dependent study, Tni media were treated with 25 μg of bromelain/mg of total protein at 37^o^C for different time points (30, 60, 120, and 240 mins). For bromelain heat inactivation assay, bromelain was heated at 80^o^C for 8 min and then treated with S-Ectodomain expressing Tni cell supernatant (25 μg of bromelain/mg of total protein) at 37^°^C for 30 min. For cysteine protease inhibitor (E64) treatment assay, bromelain plus E-64 (1, 2, and 4 µM) was used to incubate with S-Ectodomain expressing Tni cells supernatant (25 μg of bromelain/mg of total protein) at 37^°^C for 30 min. For treatment with VeroE6 cells, bromelain (75 μg/ml) plus E-64 (4μM) was incubated with VeroE6 cells at 37^°^C for 2 h. At the end of the reaction, 4X laemmli buffer was added in the mixture and boiled at 100^°^C for 5 min. The protein concentration in the cell lysate and media was measured by BCA kit (ThermoFisher, MA, USA)as per the manufacturer’s instructions.

**SDS-PAGE and Western blot analysis**

Equal concentrations of media were mixed with 4X loading buffer (Invitrogen, MA, USA) and boiled at 100°C for 5 min. The SDS-PAGE was performed in 4-20% gradient gel (Bio-Rad, CA, USA). For western blot, equal concentrations of cell lysate and media proteins were transfer into 0.45 μm PVDF membrane (Millipore, MA, USA). Further, blocked with 5% skimmed milk powder at room temperature for 1 h, the membrane was incubated with the target primary antibody with desired dilution (anti-ACE-2, 1:1000 (Cell signaling technology, MA, USA); TMPRSS2, 1:1000 (Santa Cruz Biotechnology, TX, USA) and anti-SARS Spike protein 0.05 μg/ml, (Novus Biologicals, CO, USA) at 4°C for overnight. For loading, control membranes were probed with anti-β-actin and anti-GAPDH antibodies (1:5000, Cell signaling technology, MA, USA). The membrane was washed with 1X TBST (3 X 5 min) and then incubated with respective secondary antibodies (Horse anti-mice 1:2000; Horse anti-rabbit 1:2000, (Cell signaling technology, MA, USA) and developed by using ECL chemiluminescent reagent (Bio-Rad, CA, USA).

**Bromelain treatment of SARS-CoV-2 and negative staining electron microscopy (EM)**

SARS-CoV-2 (Italy-INMI1) (*2*) was propagated in Vero E6 cell culture and harvested at day 3 post infection (MOI ~0.01). The virus suspension was inactivated with 2% paraformaldehyde in 0.05 M Hepes buffer (*3*). Bromelain (Sigma-Aldrich, Germany) was dissolved in PBS (500 µg/ml). Inactivated SARS-CoV-2 suspension was mixed with an equal volume of the Bromelain solution (250 µg/ml final concentration) and incubated for 1.5 h at 37°C, 700 rpm (Eppendorf Thermomixer compact, Germany). For negative control, inactivated SARS-CoV-2 suspension was mixed with PBS (1:1 [v/v]) and incubated as mentioned before. Since the high salt concentration of the treated suspension interferes with the negative staining procedure, a molecular centrifugation filter (30 kDa, ROTI SpinMINI; Carl Roth, Germany) was used to clean sample suspensions. Filter units were filled with 500 µl Ampuwa water (Fresenius, Germany) and centrifuged at 3000 *g* for 5 min in a swing-out centrifuge. The water in the lower reservoir remained for the next centrifugation step. Alcian blue-treated copper grids (400 mesh, coated with a formvar film) were washed on four droplets of Ampuwa water (Laue 2010), placed on a support (clipring of a Gatan 626 cryo-holder) and then on the surface of the molecular filters. 20 µl of incubated sample suspension were mixed with 160 µl Ampuwa water, filled in the prepared filter units and centrifuged at 3000 *g* for 5 min in a swing-out centrifuge. Grids were removed from the filter units, washed over three droplets of Ampuwa water and contrasted with 0.5% phosphotungstic acid (*4*). Transmission-EM was performed with a transmission electron microscope (Tecnai Spirit, Thermo Fisher Scientific) at 120 kV. Images were recorded with a side-mounted CCD camera (Megaview III, EMSIS, Germany) and 1376 x 1032 pixel.

**Protein-protein docking**

The structures of the SARS-CoV-2 S protein and the stem bromelain precursor were obtained from the RCSB Protein Data Bank, PDB codes 6VYB and 6U7D, respectively. The YASARA (www.yasara.org) homology modeling package was used to construct an atomic detail model of stem bromelain; the stem bromelain precursor was the template. The FASTA sequence used for stem bromelain was P14518 from Uniprot. Both the S protein and the bromelain homology model were imported into the Schrodinger software suite, where they were preprocessed and minimized using the protein preparation wizard. All NAG molecules were removed from the S protein structure prior to minimization. The protein-protein docking module of Schrodinger was used to dock bromelain to the S protein. 20,000 poses were generated and poses which placed the catalytic His 158 of bromelain further than 10 Å from any cysteine residue which participates in a disulfide bridge were discarded.

**Spike protein binding assay**

**Confocal microscopy:** VeroE6 (1 X 10^5^ / well) cells were seeded on coverslips in a 12 well plate. After 24 h, the cells were treated with 75 μg/ml of bromelain and mock (1XPBS) for 1 h in serum-free media. Next, the cells were washed with 1X PBS two times and incubated with SARS-CoV-2 S-Ectodomain-GFP (1 μg) in a serum-free media for 2 h. Cells were washed and fixed with 4% paraformaldehyde for 15 min. The Spike protein binding was analyzed by using a Zeiss LSM 800 confocal laser scanning microscope (UNMC Core facility). Incubation of cells with GFP-His tag protein (Sino Biological, PA, USA) served as a negative control.

**Flow cytometry:** VeroE6 cells were treated with 75 μg/ml of bromelain and mock (1XPBS) for 1 h in serum-free media. Next, the cells were detached by using 2mM EDTA in PBS and washed twice with 1X PBS. 1 X 10^6^ cells were incubated with S-Ectodomain-GFP (1 μg) in a serum-free media for 2 h. The cells were washed and resuspended in HBSS media for flow cytometry analysis at the UNMC core facility (BD LSR II flow cytometer). The results were analyzed by FlowJo software.

**SARS-CoV-2 infection assay**

VeroE6 (1x10^4^ cells) were seeded in 96 well plate. For pre-treatment with VeroE6 cells: After 24 h, cells were washed and treated with bromelain (75 μg/ml for 2 h) in a serum-free medium (n=6). For pre-treatment with SARS-CoV-2 virus: SARS-CoV-2 (MOI 0.01) incubated with medium containing bromelain (75 μg/ml) for 1 h at 37°C (n=4). For co-treatment: SARS-CoV-2 (MOI 0.01) was suspended in medium containing bromelain (75 μg/ml) and directly added to the VeroE6 cells (n=4). The incubation of cells with PBS served as a vehicle control. The pre-treated or co-treated VeroE6 cells were incubated with virus for 1 h at 37°C. After infection, cells were washed and replaced with 5% FBS containing media. After 24 h post-infection, cells were fixed with 4% buffered paraformaldehyde (Electron Microscopy Sciences) for 15 min at room temperature. The fixed cells were washed with PBS then permeabilized in 0.1% Triton X100 PBS solution for 15 min then blocked in 3% BSA PBS solution. The cells were incubated with anti-S protein Rab (Sino Biological, PA, USA) at 1:1000 in the blocking solution overnight at 4°C, followed by incubation with 1:2000 diluted Alexa Fluor 488 conjugated secondary antibody (Thermo Fisher, MA, USA) for 1 h at room temperature. The cells were counterstained for nuclei with Hoechst 33342 (Thermo Fisher, MA, USA). The fluorescent images were captured by using a Nikon Eclipse Ts2R fluorescent microscope.

**qRT-PCR assay**

**For binding:** VeroE6 (3.5 X 10^4^ / well) cells were seeded in a 12 well plate. After 24 h, the cells were pre-treated with 75 μg/ml of bromelain and vehicle (PBS) for 1 h in serum-free media. The cells were incubated with SARS-CoV-2 virus (MOI 0.01) for 1 h at 4°C. The remaining unbound/adsorbed virus was washed with PBS. The RNA was extracted from the virus infected vehicle and bromelain treated VeroE6 cells using QIAmp viral RNA isolation kit (Qiagen, MD, USA) as per the manufacturer instructions. One-step qRT-PCR (UltraPlex®1-step toughmix from Quantabio, MA, USA) was performed on isolated RNA by using 2019-nCoV_N1 kit (Biosearch technologies, CA, USA) with the following forward primer 5’ GACCCCAAAATCAGCGAAAT 3’, reverse primer 5’ TCTGGTTACTGCCAGTTGAATCTG 3’ and probe 5’ FAM-ACCCCGCATTACGTTTGGTGGACC-BHQ-1 in a CFX96 Thermal cycler (Bio-Rad, CA, USA). PCR conditions includes 50°C for 10 min followed by 95°C for 10 min and 45 cycles of 95°C for 15 sec and 60°C 1min. **For infection:** VeroE6 (3.5 X 10^4^ / well) and Calu-3 (5 X 10^4^ / well) cells were seeded in a 12 well plate. After 24 h, the cells were co-treated with 75 μg/ml of bromelain and vehicle (PBS) plus SARS-CoV-2 virus (MOI 0.01) for 1 h in serum-free media at 37°C. After incubation, the cells were replaced with media containing 5% FBS. After 24 hours of incubation, RNA was isolated from the cell culture supernatants by using QIAmp RNA isolation kit (Qiagen, MD, USA) as described above. The viral RNA was amplified by using one-step qRT-PCR as described above.

**Statistical Analysis**

Statistical analysis was performed with GraphPad Prism version 8 using a two-tailed unpaired *t*-test. Statistical significance was determined at *P* <0.05.

**KEY RESOURCES TABLE**

| **REAGENT or RESOURCE** | **SOURCE** | **IDENTIFIER** |
| --- | --- | --- |
| **Antibodies** | | |
| Polyclonal anti-ACE2 antibody | Cell signaling Technology | Cat.No: 4355 |
| Monoclonal anti-TMPRSS2 | Santa Cruz Biotechnology | Cat.No:sc-515727 |
| anti-SARS spike protein | Novus biologicals | Cat.No: NB100-56578 |
| Anti-S protein Rab | Sino Biologicals | Cat.No: 40592-R001 |
| Monoclonal anti-β-actin | Cell signaling Technology | Cat.No: 3700 |
| Monoclonal anti-GAPDH | Cell signaling Technology | Cat.No: 5174 |
| Horse anti-mouse IgG | Cell signaling Technology | Cat.No: 7076 |
| Horse anti-rabbit IgG | Cell signaling Technology | Cat.No:7074 |
| Alexa fluor 488 conjugated IgG | Thermo Fisher Scientific | Cat.No: A32731 |
| Biotin conjugated goat anti-human IgG | Abcam | Cat.No:ab64666 |
| **Cell Lines** | | |
| Vero E6 | ATCC | ATCC Cat.No:CRL-1586 |
| A549 | Laboratory of Maher Abdulla, UNMC | ATCC Cat.No:CRM-CCL-185 |
| BEAS-2B | Laboratory of Toddy Wyatt, UNMC | ATCC Cat.No:CRL-9609 |
| T3M4 | Laboratory of Michael A Hollingsworth, UNMC | Cellosaurus, Accession.No:CVCL_4056 |
| Calu3 | Laboratory of John Dickinson, UNMC | ATCC Cat.No:HTB-55 |
| HEK293T | Laboratory of Michael A Hollingsworth, UNMC | ATCC Cat.No:CRL-3216 |
| **Bacterial and virus strains** | | |
| SARS-CoV-2 | Strain:BEI_USA-wa1/2020 | NR-52281 |
| **Commercial assay kits** | | |
| Luminax xMAP antibody coupling kit | Luminex corp. | Cat.No:40-50016 |
| Magplex Microspheres | Luminex corp. | Cat.No:MC10043 |
| BestBac 2.0 Linearized Baculovirus DNA kit | Expression Systems | 91-200 |
| Pierce™ BCA Protein Assay Kit | Thermo Fisher Scientific | Cat.No:23227 |
| Deglycosylation kit | Agilent | Cat.No:GK80110 |
| QIAamp Viral RNA Mini Kit | Qiagen | Cat.No: 52904 |
| UltraPlex 1-Step ToughMix® Kit | Quantabio | Cat.No: 95166-500 |
| 2019-nCoV CDC Probe and Primer Kits for SARS-CoV-2 | Biosearch Technologies | Cat.No: KIT-NCOV-PP1-1000 |
| **Chemicals and recombinant proteins** | | |
| Bromelain | Sigma | Cat.No: B4882 |
| E-64 | Sigma | Cat.No: E3132 |
| One cOmplete ULTRA protease inhibitor tablet | Roche | Cat.No: 11697498001 |
| RIPA lysis buffer | Thermo Fisher Scientific | Cat.No: 89901 |
| 4X loading buffer | Alfa Aesar | Cat.No: J60015 |
| 4-20% gradient gel | Bio-Rad | Cat.No: 4568094 |
| 4% paraformaldehyde | Electron microscopy sciences | Cat.No: 50-980-487 |
| Econo-Pac® Chromatography Column | Bio-Rad | Cat.No: 7321010 |
| Ni sepharose excel resin | Cytiva Lifesciences | Cat.No:17-3712-01 |
| Human ACE2-AviTag protein | Acro Biosystems | Cat.No: AC2-H82F9 |
| Streptavidin R-phycoerythrin | MOSS Inc. | Cat.No: SAPE-001G75 |
| pVL1393 | Expression systems | Cat.No: 91-012 |
| GFP-His tag protein | Sino Biological | Cat.No:13105-S07E |
| Tni cells | Expression systems | Cat.No: 94-002S |
| Sf9 cells | Expression systems | Cat.No: 94-001S |
| **Software and algorithms** | | |
| DNAstar Protean software | DNAstar | https://www.dnastar.com/software/protein/ |
| Bio-Rad ProteOn Manager software(version 3.1) | Bio-Rad | https://www.bio-rad.com/en-ug/product/ |
| Flow Jo software | FlowJo, LLC | https://www.flowjo.com/ |
| Nikon NIS-Elements D software | Nikon | https://www.nikon.com/products |
| YASARA homology modeling package | YASARA | http://www.yasara.org/products.htm |
| Schrodinger software suite | Schrodinger | https://www.schrodinger.com/ |
| GraphPad Prism (8.0) | GraphPad Software | https://www.graphpad.com/ |
| Adobe photoshop CC2019 | Adobe | https://www.adobe.com/products/ |

**References:**

1. Wrapp D, Wang N, Corbett KS, et al. Cryo-EM structure of the 2019-nCoV spike in the prefusion conformation. *Science.* 2020; 13;367(6483):1260-1263.
2. Colavita F, Lapa D, Carletti F, et al. SARS-CoV-2 Isolation From Ocular Secretions of a Patient With COVID-19 in Italy With Prolonged Viral RNA Detection. *Ann Intern Med.* 2020;4;173(3):242-243.
3. Möller L, Schünadel L, Nitsche A, Schwebke I, Hanisch M, Laue M. Evaluation of virus inactivation by formaldehyde to enhance biosafety of diagnostic electron microscopy. *Viruses.* 2015;10;7(2):666-79.
4. Laue M. Electron microscopy of viruses. *Methods Cell Biol.* 2010;96:1-20.
